# PEAK1, a Novel Kinase Target in the Fight Against Cancer

**DOI:** 10.18632/oncotarget.128

**Published:** 2010-07-08

**Authors:** Jonathan A. Kelber, Richard L. Klemke

**Affiliations:** Department of Pathology and Moores Cancer Center, University of California San Diego, La Jolla, CA 92093, USA

**Keywords:** Pseudopodia Isolation, Phosphoproteomics, Non-Receptor Tyrosine Kinase, Cytoskeleton, Cell Migration, Cancer Progression

## INTRODUCTION

The completion of the human genome has enabled the identification of almost all protein kinase genes (approximately 518 total). While this number is less than originally predicted [1], it constitutes almost 2% of the entire human genome [2]. Because kinases are integral to almost all signaling processes in eukaryotic cells via their role as posttranslational modifiers, it is crucial to identify and characterize their molecular and functional properties in order to more fully understand human physiology and disease. For example, kinases play central roles in cellular processes ranging from cell-cycle progression to cell motility to cell differentiation, and their proper function controls development and homeostasis in the adult [3-8]. Additionally, dysregulation of kinase genes accounts for the origin of many human diseases, making them an important family of genes to comprehensively understand - possibly leading to novel therapeutic approaches for fighting multiple pathologies [9-13]. Well known kinases, such as Src, MAPK and, ErbB2 have well established roles during development and cancer progression. Critical insights into their functions and mechanisms of action have led to important therapeutic strategies for fighting cancer [5]. Therefore, since a significant number of novel kinases (71) remain unconfirmed at the protein level [2], it is important to gain a more complete functional understanding of the human kinome. Furthermore, many of these are likely to contribute to cancer and provide novel therapeutic targets to circumvent the side effects of current chemical and radiation therapies or the acquired resistance to more specific therapies.

Over the past decade, our lab has developed a number of new approaches for studying the role of the cellular cytoskeleton and the subcellular domains that it regulates during cell migration, neurogenesis and cancer progression [14-22]. More specifically, we have developed a unique method to purify and characterize cell pseudopodia using a 3.0 μm porous membrane system. When attached to the top of these membranes and subjected to a chemoattractant, cells begin to protrude their pseudopodia through these pores - they can then be mechanically isolated from the remainder of the cell body [19-21]. We used these unique approaches to probe the proteome and phosphoproteome of these subcellular structures. Our ability to affinity purify pY proteins from isolated pseudopodia proved to be a robust system to identify proteins involved in cell migration. Also, the complete solublization of pseudopodial proteins in SDS buffer significantly improved our yield of pY proteins, which can be tightly associated with the insoluble cytoskeleton [23]. This approach allowed us to identify many low abundance pY proteins involved in cell migration and led to the discovery of the phosphotyrosine protein and kinase, PEAK1 (pseudopodium-enriched atypical kinase 1, KIAA2002, sgk269). Collectively, our findings demonstrate that PEAK1 is a new non-receptor tyrosine kinase that operates within Src-p130Cas-Crk-Paxillin and Ras-Raf-Erk signaling pathways to regulate cell proliferation, migration and cancer progression [24] (Table 1).

**Table 1:** Summary of known PEAK1 functions. NT = not tested.

Function	Domain	Residues	Molecular and Cancer Implications
Crk Binding	C-terminal	P1153	May help recruit Crk into p130Cas/Crk complex to induce cell motility; may lead to metastasis
Src Substrate and Binding	N-terminal	Y665	Tyrosine phosphorylation of PEAK1 may activate a positive feedback loop between Src and PEAK1 allowing for further p130Cas/Crk coupling; may promote or be required for potent Src-transforming functions
Ubiquitously Expressed	NT	NT	Upregulation or mutational activation may drive tumor progression
Actin Localization	N-terminal	a.a. 339-727	May facilitate actin remodeling or the recruitment of actin remodeling molecules to the cytoskeleton to enable cell motility and cancer cell metastasis
Focal Adhesion Localization	NT	NT	May act to recruit p 130Cas/Crk and/or Paxillin to focal adhesions; may also function in focal adhesion dynamics, adhesion maturation and/or cell detachment during metastasis
Tyrosine Kinase	C-terminal	a.a. 1330-1664	Likely functions to phosphorylate multiple substrates during normal homeostasis and tumor progression
Phosphosites (Predicted/Known)	N- and C- terminal	Y (387, 475, 531, 596, 618, 636, 641, 665, 979, 880, 1107, 1153, 1348, 1372) S/T (779, 783)	May provide docking sites for other molecules (e.g. Erk, p130Cas, Crk and Src) or alter molecular confirmation to induce activation; kinase or scaffolding functions in these pathways are likely to contribute to their already well-established role during cancer progression

**Fig. 1.**
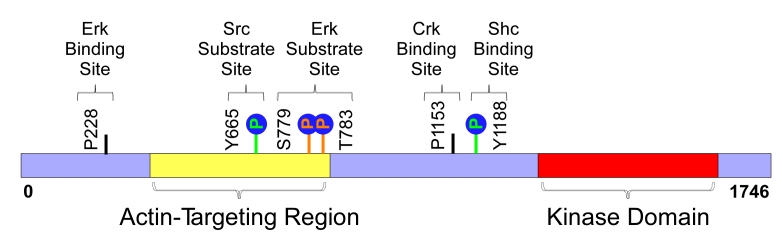
A schematic showing PEAK1 actin-targeting and kinase domains as well as the predicted binding or substrate residues for Src, Erk, Crk and Shc proteins.

PEAK1 is a member of the new kinase family three (NKF3) and its domain structure is complex, with predicted consensus binding and/or substrate sites for Src, Erk, Shc and Crk. Notably, PEAK1 is ubiquitously expressed in multiple tissues, suggesting that it has a major role in normal physiology [24]. In addition, we determined that PEAK1 contains an Nterminal actin-targeting region and a functional Cterminal atypical kinase domain (Figure 1). All active kinases are predicted to contain three motifs VAI**K**, HR**D**, and **D**FG within the kinase domain [2]. Each motif contains one highly conserved residue (VAI**K**: **K**, HR**D**: **D**, **D**FG: **D**) that is predicted to be important for full catalytic activity. Sequence analysis revealed that PEAK1 contains all three motifs YAVK, HCD, and NFS. While the YAVK and HCD motifs are highly conserved on the critical **K** and **D** residues, the **D** residue in the NFS motif is replaced by N, which classifies it as an atypical kinase [2]. However, little is known about whether this amino acid substitution can affect kinase catalytic activity or whether this PEAK1 residue (and/or others) may be mutated in human cancers to confer full catalytic activity [2]. While we were initially able to demonstrate tyrosine kinase activity in the full-length protein that had been immunoaffinity purified from mammalian cells, this did not remove the possibility that other tyrosine kinases bind and co-precipitate with PEAK1, accounting for the detected activity. To address this possibility, we generated a C-terminal construct that included the kinase domain (a.a. 1289-1746) that was expressed in and purified from E. coli. Notably, this allowed us to generate large amounts of the purified PEAK1 truncation mutant, and it also displayed intrinsic tyrosine kinase activity *in vitro* [24].

In agreement with PEAK1 containing an actintargeting region, modulation of PEAK1 protein levels affected the phosphorylation level of several known cytoskeletal regulatory proteins including paxillin, p130Cas and Erk, and it was found to associate with the Crk adaptor protein. Crk regulates cell spreading and migration by coupling critical signaling proteins such as EGFR, ErbB2, PDGFR, C-Abl and p130Cas to the cytoskeleton and focal adhesions [25]. p130Cas and its family members are necessary for cell migration and cancer progression *in vitro* and *in vivo* [26-28]. The Src-p130Cas-Crk complex has been shown to modulate Rac activity, pseudopodium protrusion, cell migration and cancer progression [27-30]. We have also shown that Src is necessary for growth factor- and integrininduced tyrosine phosphorylation of PEAK1 [24]. Together, these findings suggest that integrins and growth factors may cooperate to activate Src, which in turn phosphorylates p130Cas on Y165, Y249, or Y410 leading to Crk binding through its SH2 domain. Via its association with the SH3 domain of Crk, we predict that PEAK1 assists in the recruitment of Crk to the Srcp130Cas- Crk scaffold. PEAK1's subsequent phosphorylation by Src on Y665 may function to facilitate its cytoskeletal or focal adhesion localization and/or its kinase activation. It is very likely that these PEAK1-mediated molecular interactions are critical for the proper dynamic rearrangement of the actin cytoskeleton and focal adhesions during cell migration, as cells undergo a significant reduction in their migratory potential when depleted of PEAK1 protein [24]. It will be important for future studies to characterize the role of PEAK1 during early, proliferative versus late, metastatic stages of cancer progression. Nevertheless, PEAK1 likely fulfills several important molecular functions. First, it may modulate protein-protein interactions by directly phosphorylating components of the Src-p130Cas-Crk scaffold via its tyrosine kinase activity. Second, given that PEAK1 translocates to focal adhesions and the actin cytoskeleton, it could provide a mechanism to transport the Src-p130Cas-Crk scaffold to these structures. Finally, it may deliver unique effector proteins to these cytoskeletal structures, which in turn may regulate cell migration and/or proliferation. In any case, our observations that PEAK1 can interact with and modulate the Src-p130Cas-Crk-Paxillin and Ras-Raf-Erk pathways downstream of integrins and receptor tyrosine kinases (RTKs) point to a central role for this novel tyrosine kinase during cell migration and proliferation in normal and transformed cells [24].

Although we are the first to clone and directly study the function of PEAK1, several independent lines of evidence also suggest that PEAK1 and its only family member sgk223 (pragmin) (33% overall homology to PEAK1) play integral roles in regulating cell motility and tumor progression [31-32]. For example, sgk223 has been reported to be a novel effector of Rnd2 GTPase, and has also been shown to stimulate RhoA activity in HeLa cells and mediate cancer cell invasion in a Src-dependent manner [32]. More recently, sgk223 and PEAK1 were both identified in a quantitative phosphoproteomics study as potential targets that mediate Src-induced invasion in advanced colon carcinoma cells [31]. We have recently demonstrated that PEAK1 promotes proliferation, migration and anchorage-independent growth *in vitro* and tumor formation *in vivo* [24]. While large shRNA genomic screens have suggested that PEAK1 is involved in cancer cell proliferation [33], our recent report is the first to directly link PEAK1 function with cancer progression and demonstrate that PEAK1 levels are amplified in over 80% of colon cancer patients (primary and metastatic lesions). Notably, this underscores the importance of understanding the role of PEAK1 in human cancers (Table 1).

**Fig. 2:**
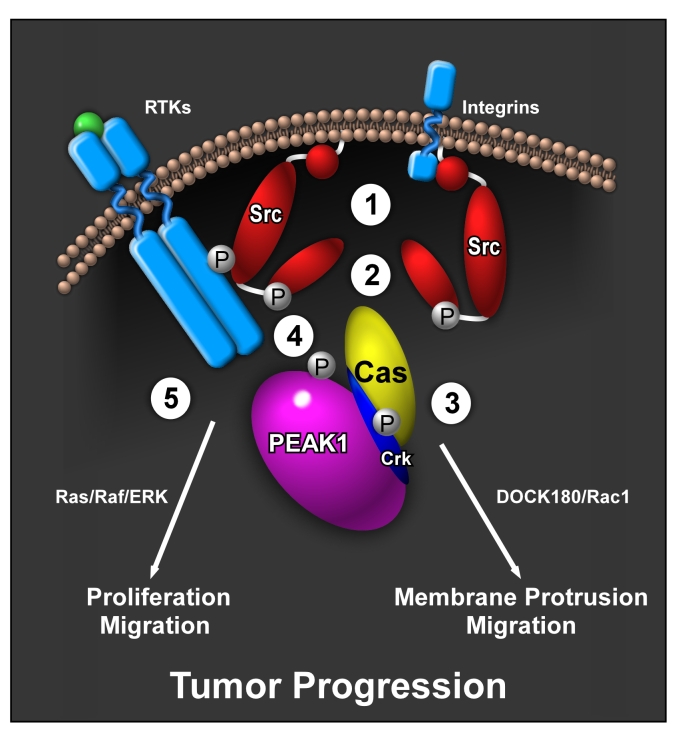
A model depicting the role of PEAK1 in mediating signaling events through the Src-p130Cas-Crk and Ras-Raf-Erk signaling pathways downstream of receptor tyrosine kinases (RTKs) and integrins. It is likely that PEAK1 has both scaffolding and catalytic functions in these pathways.

Predicted PEAK1-mediated steps during RTK- or Integrin-induced tumor progression:

1. Ligand/RTK- or Integrin-induced binding of activated Src (Y416).
2. Src-mediated tyrosine phosphorylation of p130Cas (Y165, Y249, Y410, etc.).
3. Binding of Crk (SH3 domain) to PEAK1 (P1153) and recruitment of the PEAK1/Crk complex to phosphop130Cas - PEAK1 enables multiple Crk molecules to bind to multiple phosphotyrosine residues on p130Cas. Enhanced Crk activation and binding to DOCK180 leads to Rac1 activation, membrane protrusion and cell migration.
4. Src-mediated tyrosine phosphorylation of PEAK1 (Y665) leads to enhanced and/or sustained Src phosphorylation (Y416) and activation (possibly through PEAK1 tyrosine kinase activity) - this further enhances 130Cas/Crk coupling and the subsequent signaling events downstream of Crk activation.
5. PEAK1 may also promote cell proliferation and migration through the Ras/Raf/Erk pathway downstream of RTKs and Integrins, via its predicted Erk binding (P228) and substrate (S779/T783) sites.

While it is clear now that PEAK1 has intrinsic tyrosine kinase activity *in vitro*, future studies should probe the role of its kinase domain during tumor progression and identify potential substrates of this novel cytoskeleton-associated kinase. More comprehensive mechanistic studies are also warranted in order to more completely understand how PEAK1 couples to multiple intracellular signaling pathways and the actin cytoskeleton or focal adhesions to promote cell proliferation and motility. Specifically, it will be crucial to elucidate the differential roles for PEAK1 in the Src-p130Cas-Crk-Paxillin and Ras-Raf- Erk signaling pathways. Finally, understanding the upstream regulators of PEAK1 and why it is upregulated in human colon cancers (and whether it is also upregulated in other human cancers) will likely help clinical oncologists understand disease progression. Importantly, PEAK1 is a critical regulator of multiple cellular processes and its tyrosine kinase domain may likely provide a novel therapeutic target for controlling aberrant cellular functions that lead to cancer.
